# Rare Diseases on the Internet: An Assessment of the Quality of Online Information

**DOI:** 10.2196/jmir.7056

**Published:** 2017-01-18

**Authors:** Frédéric Pauer, Svenja Litzkendorf, Jens Göbel, Holger Storf, Jan Zeidler, Johann-Matthias Graf von der Schulenburg

**Affiliations:** ^1^ Center for Health Economics Research Hannover (CHERH) Leibniz University Hannover Hannover Germany; ^2^ Medical Informatics Group (MIG) University Hospital Frankfurt Frankfurt am Main Germany

**Keywords:** health literacy, rare disesases, quality indicators, health information exchange

## Abstract

**Background:**

The importance of the Internet as a medium for publishing and sharing health and medical information has increased considerably during the last decade. Nonetheless, comprehensive knowledge and information are scarce and difficult to find, especially for rare diseases. Additionally, the quality of health or medical information about rare diseases is frequently difficult to assess for the patients and their family members.

**Objective:**

The aim of this study is to assess the quality of information on the Internet about rare diseases. Additionally, the study aims to evaluate if the quality of information on rare diseases varies between different information supplier categories.

**Methods:**

A total of 13 quality criteria for websites providing medical information about rare diseases were transferred to a self-disclosure questionnaire. Identified providers of information on the Internet about rare diseases were invited to fill out the questionnaire. The questionnaire contained questions about the information provider in general (eg, supplier category, information category, language, use of quality certificates, and target group) and about quality aspects that reflect the 13 quality criteria. Differences in subgroup analyses were performed using *t* tests.

**Results:**

We identified 693 websites containing information about rare diseases. A total of 123 questionnaires (17.7%) were completely filled out by the information suppliers. For the remaining identified suppliers (570/693, 82.3%), the questionnaires were filled out by the authors based on the information available on their website. In many cases, the quality of websites was proportionally low. Furthermore, subgroup analysis showed no statistically significant differences between the quality of information provided by support group/patient organization compared to medical institution (*P*=.19). The quality of information by individuals (patient/relative) was significantly lower compared to information provided by support group/patient organization (*P*=.001), medical institution (*P*=.009), and other associations and sponsoring bodies (*P*=.001) as well.

**Conclusions:**

Overall, the quality of information on the Internet about rare diseases is low. Quality certificates are rarely used and important quality criteria are often not fulfilled completely. Additionally, some information categories are underrepresented (eg, information about psychosocial counseling, social-legal advice, and family planning). Nevertheless, due to the high amount of information provided by support groups, this study shows that these are extremely valuable sources of information for patients suffering from a rare disease and their relatives.

## Introduction

The quality of information provided on the World Wide Web has been highly discussed in the literature for the past few years (eg, [1-3]). In particular, regarding medical information, the provision of high-quality information is very important because misinformation can lead to serious health consequences for the affected patients. This is particularly relevant for information on the World Wide Web, where the information is used without the intervention of a medical professional, even though the related websites clearly state that this information cannot replace a medical professional’s consultation [4-9].

In the field of rare diseases, information is scarce; it is difficult to find the right information as well as to assess the quality of the provided information in detail [10-12]. Additionally, only a few medical experts for specific rare diseases have comprehensive knowledge about the diseases. This limits the ability of patients to get access to high-quality information [13,14]. The definitions of rare diseases vary from 12:100,000 in Australia to 75:100,000 in the United States [15]. This study is set in Germany; therefore, it is based on the European Union definition that considers diseases to be rare when the prevalence is less than 50:100,000 [16]. It is estimated that there are between 5000 and 8000 different rare diseases affecting nearly 30 million people in the European Union and 4 million people in Germany alone [15,17,18].

A detailed description of the framework of this study can be found in the literature [19]. In brief, the aim of the project is to conceptualize and implement a central information portal about rare diseases in Germany, which refers to existing quality-assured information sources [20]. The distribution of information and knowledge about rare diseases is an important factor to improve the overall situation of people affected by a rare disease [17,21]. In this context, the Internet as a worldwide open-access medium has become more important during the last decade [22,23]. The Internet can improve the distribution of information about rare diseases to the general public and, in particular, to medical professionals, patients, and relatives of patients [22]. For the latter group, the Internet is one of the most frequently used information resources and often the primary source to search for information after getting a diagnosis [24]. Nevertheless, patients reported that they are often overstrained with the information they find on the Internet [25]. Information is often disordered and refers to different stages of the disease. Moreover, it is not possible to assess the quality of the information and to find the right information, such as social-legal advice [1]. For medical professionals, it is important to have access to the latest innovative research results and evidence-based therapeutic options as well as actual contact details of support groups [26].

The aim of this study is to assess the quality of information on the Internet about rare diseases. Additionally, the study aims to evaluate if information about rare diseases (eg, information provided by support groups) is as reliable as information provided by medical institutions by performing subgroup analyses. The assessment is based on 13 quality criteria for websites providing medical information about rare diseases [19].

## Methods

We divided the methodological framework into several steps. First, as mentioned previously, 13 quality criteria for websites providing medical information were included to a self-disclosure questionnaire. The questionnaire contained questions about the information provider in general (eg, supplier category, information categories, language, use of quality certificates, and target group) and questions about quality aspects reflecting the 13 quality criteria (Textbox 1). The disclosure was not anonymous because the answers need to be checked by the authors. The questionnaire was verified and pretested by the patient organization Alliance of Rare Chronic Diseases Germany (ACHSE eV) and Orphanet Germany. Additionally, the verified version of the questionnaire was tested by selected rare disease information providers, which were randomly identified by an Internet search.

Second, information providers on the Internet were identified by an Internet search; all 8000 rare diseases, as listed in the Orphanet list of rare diseases and synonyms [27], were entered into the Google search engine by a number of research assistants from May 2015 to January 2016. This list included all registered rare diseases and their synonyms. For every disease, the first two hit lists, meaning the first 20 hits, were screened to identify information websites in the German language. A random check with 30 diseases showed that we could assume that a screening of the first two hit lists of each rare disease was sufficient to identify all relevant information websites. Websites that provided information about rare diseases were included in the database, whereas those that just presented contact data, for example, with no further information were excluded. Furthermore, websites providing information about several rare diseases were included into the database as a singular information provider. Third, all information providers were invited by email to fill out the self-disclosure questionnaire (September 2015 to March 2016). Then, these datasets were double-checked using the information available on the website. Data were checked for correctness (eg, does the website provide information about the stated information category?) and plausibility (eg, is the description of the process of systematic or literature research comprehensible?). For all information providers who did not fill out the questionnaire, the questions were answered by the authors based on the information available on the website. For that, authors checked the content and the characteristics of each identified website carefully. However, just 10 of 13 quality criteria could be answered by publicly available information. The remaining three quality criteria, representing the authoring information, evaluation of information, and review of information, were not reviewable by the authors. Consequently, for the main evaluation, these quality criteria were excluded. In the end, all datasets were evaluated. Microsoft Access was used for data storage. For data analysis, both Microsoft Excel and Microsoft Access (versions 2007) were used. Differences in subgroup analyses were performed using *t* tests.

**Quality criteria for websites about rare diseases.**

**Authoring information**

- Do you perform a systematic (literature) search prior to providing information for your home page? If yes, then please describe this process.
- Are experts involved in providing information? If yes, then which field do they belong to?
- Do you document the process of providing information? If yes, then please describe the documentation process.
- Do you inform users about the process of developing information? If yes, please describe the process and provide the respective URL.

**Authors**

- Is general information about the authors mentioned?
- Are other persons who contributed to developing information mentioned?
- Is user-generated content distinguishable and labeled with a username?

**Sources**

- Does the information concern primary sources of information?
- If no, then do you quote external sources?

**Creation or update date**

- Is the creation date of information mentioned?
- Is the update date of information mentioned?

**Privacy statement**

- Is a privacy policy used to inform the user about the usage, storage, and disclosure of personal data?
- Do you inform the user in a prominent position about the storage of personal data for internal usage (eg, research) with an analysis tool and does the user has the opportunity to disagree?
- Does the user has to agree actively to the disclosure of personal data to third parties?

**Declaration of evidence**

- Is all medical information evidence-based and it is discernible on what basis points are made (eg, studies, expert statements)?
- Do you provide references to the limitations of the evidence and set out further evidence needs?

**Marking of conflicts of interests**

- Are advertisements marked as such plainly?
- Are sponsors named?
- Are targets and purposes of the home page published (eg, commercial interest)?
- Is the funding (except from self-financing) published?
- Are conflicts of interests mentioned?

**Consideration of target group**

- Is information presented target group-specific?
- Is it discernible to whom the information is addressed (eg, patients, doctors)?

**Evaluation of information**

- Does an archive with former or changed contents exist?
- Is all information checked consistently regarding correctness and accuracy?

**Review of information**

- Does an internal review process (content quality assessment) for the evaluation of contents exist? If yes, then please describe the process.

**Characteristics of the website (accessibility)**

- Did you check the website for accessibility through a BITV-Test? (The BITV-Test is a comprehensive accessibility evaluation instrument.) If yes, how many points has the website scored in this test?
- Is the font size of the website adjustable?
- Do you consider persons with color vision deficiency in the website coloration?
- Is the main menu selectable without a mouse?
- Information is available in a simple language (eg, according to the rules of the network Simple Language).
- Is the website’s content readable by a software tool?
- Is it possible to subscribe to a newsletter?
- Is information available in a printed version?
- Are the contents shown in multimedia (eg, in terms of videos and photos)?

**Imprint**

- Is the imprint created according to § 5 TMG/§ 55 RStV following German law?

**Contact facility**

- Do users have the facility to provide feedback or to get in touch with the operator?
- Is a contact sheet easy to access?

## Results

Overall, we identified 693 information suppliers on the Internet providing information about rare diseases in the German language or from German-speaking countries. A total of 123 questionnaires (17.7%) were completely filled out by the information suppliers. For the remaining identified suppliers (570/693, 82.3%), the questionnaires were filled out by the authors, omitting the questions referring to quality criteria representing the authoring information, evaluation of information, and review of information. A list of the identified information supplier is available from the corresponding author on reasonable request.

Most of the websites were located in Germany (632/693, 91.2%), Austria (21/693, 3.0%), or Switzerland (40/693, 5.8%); therefore, most of the sites were available in the German language (682/693, 98.4%). However, some were available only, or additionally, in the English language (108/693, 15.6%). The fact that websites can be available in more than one language has to be taken into account. The majority of websites were those of patient organizations or support groups (269/693, 38.8%). Other important providers were medical institutions (186/693, 26.8%), other associations and sponsoring bodies (65/693, 9.4%), and individuals (eg, patient/relative; 52/693, 7.5%). The three most frequent information categories of all information suppliers were information about disease patterns/symptoms (633/693, 91.3%), information about diagnostics (517/693, 74.6%), and information about medication, curative means, and aids (359/693, 51.8%). Little information was available about psychosocial counseling (49/693, 7.1%), in particular. As a target group, adults were most frequently addressed (662/693, 95.5%). All characteristics are shown in detail in Table 1.

Tables 2 and 3 show the comparison and distribution between supplier and information categories. For instance, it can be seen that information provided by individuals mostly focused on disease patterns/symptoms, wherby information provided by medical institutions additionally focused on diagnostics. Furthermore, information exchange with other patients and information about psychological counseling were mostly provided by support groups/patient organizations.

**Table 1:** Characteristics of information providers (N=693).

Item		n (%)
**Supplier category**		
	Support group/patient organization	269 (38.8)
	Medical institution	186 (26.8)
	Other associations and sponsoring bodies	65 (9.4)
	Individual (patient/relative)	52 (7.5)
	Expert association	40 (5.8)
	Individual (medical expert)	29 (4.2)
	Pharmaceutical or medical technology company	26 (3.8)
	Publishing or media company	21 (3.0)
	Other	5 (0.7)
**Information category (multiple answers possible)**		
	Disease pattern/symptoms	633 (91.3)
	Diagnostics	517 (74.6)
	Medication, curative means, and aids	359 (51.8)
	Assistance for self-help	347 (50.1)
	Information exchange with other patients	320 (46.2)
	Other therapy options	317 (45.7)
	Research	254 (36.7)
	Personal advice	164 (23.7)
	Training and continued education	128 (18.5)
	Advice from doctors	116 (16.7)
	Therapeutic guidelines	101 (14.6)
	Desire to have children/family planning	93 (13.4)
	Social-legal advice	86 (12.4)
	Psychosocial counseling	49 (7.1)
**Language (multiple answers possible)**		
	German	682 (98.4)
	English	108 (15.6)
**Country**		
	Germany	632 (91.2)
	Switzerland	40 (5.8)
	Austria	21 (3.0)
**Target group (multiple answers possible)**		
	Adults	662 (95.5)
	Children	235 (33.9)
	Medical professionals	221 (31.9)
**Self-disclosure**		
	Accomplished by the supplier	123 (17.7)
	Accomplished by authors	570 (82.3)

**Table 2:** Comparison and distribution between supplier (individual-medical expert, individual-patient/relative, expert association, medical institution, and pharmacetuical or medical technology company) and information categories.

Category	Supplier									
	Individual (medical expert)		Individual (patient/relative)		Expert association		Medical institution		Pharmaceutical or medical technology company	
	n (%)	Supplier %	n (%)	Supplier %	n (%)	Supplier %	n (%)	Supplier %	n (%)	Supplier %
Medication, curative means, and aids	12 (3.3)	41.4	26 (7.2)	50.0	18 (5.0)	45.0	79 (22.0)	42.5	22 (6.1)	84.6
Information exchange with other patients	8 (2.5)	27.6	41 (12.8)	78.9	6 (1.9)	15.0	8 (2.5)	4.3	3 (0.9)	11.5
Diagnostics	22 (4.3)	75.9	27 (5.2)	51.9	30 (5.8)	75.0	158 (30.6)	85.0	21 (4.1)	80.8
Research	11 (4.3)	37.9	11 (4.3)	21.2	20 (7.8)	50.0	92 (36.2)	49.5	5 (2.0)	19.2
Training and continued education	6 (4.7)	20.7	3 (2.3)	5.8	13 (10.2)	32.5	46 (35.9)	24.7	0 (0.0)	0.0
Assistance for self-help	9 (2.6)	31.0	21 (6.1)	40.4	17 (4.9)	42.5	32 (9.2)	17.2	11 (3.2)	42.3
Desire to have children/family planning	5 (5.4)	17.2	6 (6.5)	11.5	1 (1.1)	2.5	14 (15.1)	7.5	5 (5.4)	19.2
Disease pattern/symptoms	28 (4.4)	96.6	47 (7.4)	90.4	32 (5.1)	80.0	165 (26.1)	88.7	23 (3.6)	88.5
Personal advice	6 (3.7)	20.7	2 (1.2)	3.9	7 (4.3)	17.5	41 (25.0)	22.0	5 (3.0)	19.2
Psychosocialcounseling	0 (0.0)	0.0	0 (0.0)	0.0	2 (4.1)	5.0	10 (20.4)	5.4	0 (0.0)	0.0
Other therapy options	16 (5.0)	55.2	27 (8.5)	51.9	15 (4.7)	37.5	98 (30.9)	52.7	11 (3.5)	42.3
Social-legal advice	1 (1.2)	3.5	2 (2.3)	3.9	3 (3.5)	7.5	13 (15.1)	7.0	4 (4.7)	15.4
Therapeutic guidelines	6 (5.9)	20.7	3 (3.0)	5.8	10 (9.9)	25.0	26 (25.7)	14.0	2 (2.0)	7.7
Advice from doctors	4 (3.4)	13.8	0 (0.0)	0.0	18 (15.5)	45.0	62 (53.5)	33.3	5 (4.3)	19.2

**Table 3:** Comparison and distribution between supplier (support group/patient organization, publishing or media company, other associations and sponsoring bodies, and other) and information categories.

Category	Supplier							
	Support group/patient organization		Publishing or media company		Other associations and sponsoring bodies		Other	
	n (%)	Supplier %	n (%)	Supplier %	n (%)	Supplier %	n (%)	Supplier %
Medication, curative means, and aids	148 (41.2)	55.0	15 (4.2)	71.4	36 (10.0)	55.4	3 (0.8)	60.0
Information exchange with other patients	227 (70.9)	84.4	3 (0.9)	14.3	24 (7.5)	36.9	0 (0.0)	0.0
Diagnostics	193 (37.3)	71.8	20 (3.9)	95.2	42 (8.1)	64.6	4 (0.8)	80.0
Research	76 (29.9)	28.3	4 (1.6)	19.1	33 (13.0)	50.8	2 (0.8)	40.0
Training and continuededucation	41 (32.0)	15.2	3 (2.3)	14.3	16 (12.5)	24.6	0 (0.0)	0.0
Assistance for self-help	223 (64.3)	82.9	4 (1.2)	19.1	29 (8.4)	44.6	1 (0.3)	20.0
Desire to have children/family planning	51 (54.8)	19.0	5 (5.4)	23.8	6 (6.5)	9.2	0 (0.0)	0.0
Disease pattern/symptoms	259 (40.9)	96.3	21 (3.3)	100.0	53 (8.4)	81.5	5 (0.8)	100.0
Personal advice	91 (55.5)	33.8	0 (0.0)	0.0	12 (7.3)	18.5	0 (0.0)	0.0
Psychosocial counseling	33 (67.4)	12.3	0 (0.0)	0.0	4 (8.2)	6.2	0 (0.0)	0.0
Other therapy options	108 (34.1)	40.2	14 (4.4)	66.7	24 (7.6)	36.9	4 (1.3)	80.0
Social-legal advice	54 (62.8)	20.1	0 (0.0)	0.0	9 (10.5)	13.9	0 (0.0)	0.0
Therapeutic guidelines	37 (36.6)	13.8	7 (6.9)	33.3	10 (9.9)	15.4	0 (0.0)	0.0
Advice from doctors	9 (7.8)	3.4	3 (2.6)	14.3	14 (12.1)	21.5	1 (0.9)	20.0

As a first investigation, all identified websites about rare diseases were analyzed for the use of quality certificates. The majority of websites about rare diseases did not use certifications or quality seals. Of the 693 websites analyzed, only 28 (4.0%) were certified by the international Health on the Net Foundation Code of Conduct (HONcode) [28]. Additionally, some were certified by the German certification programs German Action Forum Health Information System (afgis) [29] (7/693, 1.0%) or Medisuch [30] (8/693, 1.2%).

Table 4 shows the results for the evaluation of the quality of information on the Internet about rare diseases. The quality criteria authoring information, evaluation of information, and review of information were based on the datasets from the 123 questionnaires that were filled out by the information supplier. All other quality criteria were based on the datasets of all information providers. It was examined whether the information of websites satisfied the defined quality categories. For some categories, it was not necessary to meet every corresponding item; it was sufficient to fulfill a part of the corresponding items (eg, to fulfill the category sources, the website must contain either primary information or mention external sources, not necessarily both of them). None of the websites fulfilled all the quality criteria and the corresponding categories completely.

**Table 4:** Quality of information websites (N=693).

Item			n (%)
**Quality criteria**			
	Authoring information^a^		102 (82.9)
	Authors		376 (54.3)
	Sources		229 (33.0)
	Creation or update date		467 (67.4)
	Privacy statement		474 (68.4)
	Declaration of evidence		360 (51.9)
	Marking of conflicts of interests		211 (30.4)
	Consideration of target group		643 (92.8)
	Evaluation of information^a^		99 (80.5)
	Review of information^a^		47 (38.2)
	**Characteristics of the website (accessibility)**		
		BITV-Test (barrier-free information technology regulation)	0 (0.0)
		Font size adjustable	692 (99.9)
		Consideration of persons with color vision deficiency in coloration	396 (57.1)
		User can have read out website’s content	692 (99.9)
		Main menu selectable without a mouse	689 (99.4)
		Information in simple language	0 (0.0)
		Newsletter	120 (17.3)
		Printed version	218 (31.5)
		Multimedia	299 (43.1)
	Imprint		638 (92.1)
	Contact facility		687 (99.1)
**Use of quality certificates**			
	HONcode		28 (4.0)
	Medisuch		8 (1.2)
	Afgis		7 (1.0)
	Stiftung Gesundheit		0 (0.0)

^a^ Based on the datasets from the 123 questionnaires that were filled out by the information supplier.

More than 90% of the information suppliers fulfilled the quality criteria of providing contact facility (687/693, 99.1%), imprint (638/693, 92.1%), and consideration of target group (643/693, 92.8%). Although important quality criteria for websites providing information about rare diseases, the criteria declaration of creation or updating date (467/693, 67.4%) and privacy statement (474/693, 68.4%) were met by only approximately 70% of the identified information suppliers.

The information criteria about characteristics of the website (accessibility) can be divided into several aspects for more detailed analyses. For instance, 43.1% (299/693) of the websites provided the information with the support of multimedia, 31.5% (218/693) also provided printed information, and 17.3% (120/693) provided an email newsletter service. Moreover, 57.1% (396/693) considered persons with color vision deficiency in designing their websites. Detailed results are shown in Table 4.

**Figure 1:**
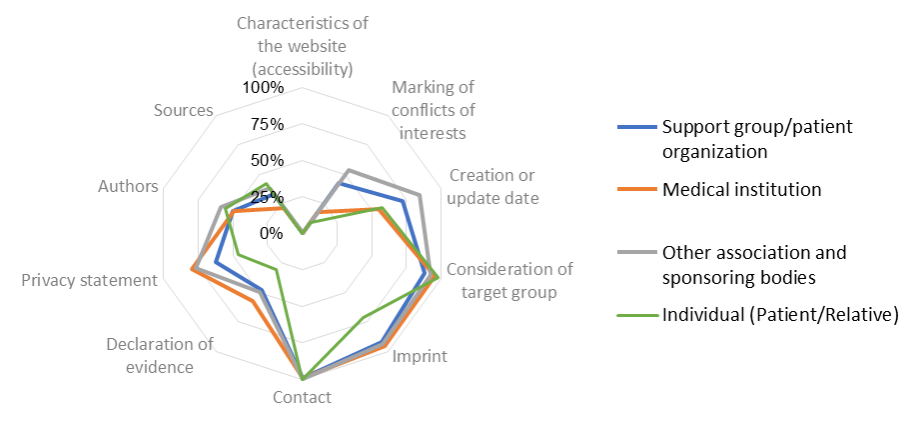
Fulfilment of quality criteria by information provider.

Subgroup analyses were performed for the four most frequent information supplier categories: support group/patient organization, medical institution, other associations and sponsoring bodies, and individuals (patient/relative). Under the assumption that the fulfillment of every single quality criterion has equal weight, the quality of information of various information supplier categories were compared. On the basis of the 10 quality categories which could be evaluated for all information providers, statistically significant differences could be observed for the supplier category individuals (patient/relative) using a *t* test analysis. The quality of information by these suppliers was significantly lower compared to information provided by support group/patient organization (*P*=.001), medical institution (*P*=.009), and other associations and sponsoring bodies (*P*=.001) as well. No statistically significant differences were observed for the quality of information provided by support group/patient organization compared to medical institution (*P*=.19). Additionally, information provided by other associations and sponsoring bodies showed statistically significant differences compared to that provided by support group/patient organization (*P=*.007) and by medical institution (*P*=.001). The quality of information provided by other associations and sponsoring bodies was significantly higher. Figure 1 shows the distribution of fulfillment of quality criteria by information and supplier categories.

## Discussion

### Principal Findings

Information about rare diseases is scarce. In the German-speaking setting, 693 websites containing information about rare diseases were identified. In many cases, the quality of these websites, based on the defined quality criteria for websites containing information about rare diseases, can be assessed as insufficient. In addition, quality certificates are rarely used by information providers of rare diseases.

Particularly, the accessibility of the websites needs to be improved, although because of browser configuration, the adjustment of the font size, the selection of the main menu without a mouse, and the readout of website’s content seems to be working for most of the websites without any problems. However, providing information by other means, such as email, newsletters, and printed versions, is offered only by some information providers. Support group/patient organizations and other associations and sponsoring bodies are more commonly among those who provide access to their information in various ways. None of the information suppliers provide information in simple language according to the official rules of the network of simple language [31]. Additionally, mentioning of sources of information and disclosing conflicts of interests are seldom stated, although these are important aspects for assessing medical or health information. Furthermore, because of rapid advantages in the development of information and to demonstrate the latest research findings, the documentation of the creation or updating date and the declaration of evidence should be stated more often. On the positive side, an opportunity to contact the website operator is provided in most cases.

Not all information suppliers provide an adequate imprint and privacy statement, even though this is required by German law. In particular, support groups/patient organizations and individuals (patient/relative) do not provide these kinds of information, although their implementation should be rather straightforward. It can be hypothesized that ignorance and limited experience prevent these supplier categories presenting themselves as professionally as other information providers online. A guidance document for support groups/patient organizations and individuals could help to improve the website’s quality.

By far, support groups and patient organizations provide most of the information websites for rare diseases. This reflects the importance of support groups for patients suffering from rare diseases and their relatives [32]. Due to limited knowledge about the diseases, the insufficient experiences of most of the medical professionals, and often limited therapeutic approaches, as well as the low number of affected patients, support groups for patients with rare diseases are important possibilities to share knowledge, experiences, and advice with other affected patients. Support groups and patient organizations for rare diseases constitute very important sources of information about rare diseases and contain high potential to solve upcoming research questions [32]. Moreover, the significant number of identified websites by individuals providing information about specific rare diseases shows that these persons feel isolated with the disease and that they want to make information about themselves public to get in touch with other people affected by the disorder.

Information about psychosocial counseling and the desire to have children and/or family planning are rarely presented on the websites containing information about rare diseases. Nevertheless, both are important information categories for patients suffering from a rare disease [26,33] and their relatives because 80% of all rare diseases have genetic causes [18]. Genetic questions are in line with questions about family planning and genetic theory. Moreover, because of the low number of affected persons and the feeling of being overstrained with the situation of being the only person suffering from this specific disease, psychosocial counseling constitutes an important role for all patients. For this, support groups and patient organizations already provide most of the available information in the categories of information exchange with other patients, assistance for self-help, family planning, personal advice, psychosocial counseling, and social-legal advice. Nevertheless, information and knowledge about psychosocial counseling and family planning in the field of rare diseases need to be extended.

Interestingly, there were no statistically significant differences identified between the quality of information provided by support groups/patient organizations and medical institutions. Only the quality of information provided by other associations and sponsoring bodies showed statistically better results than information provided by self-help group/patient organizations and medical institutions. Overall, cooperation and information transfer between all supplier categories can help to improve information quality and information access for patients suffering from rare diseases, their relatives, and medical professionals. Especially for rare diseases, cooperation activities can improve evidence-based clinical and health care research.

Future research on the quality of information about rare diseases must be considered in a more international context. Especially for ultrarare diseases, for which limited information is available and only a few people worldwide are affected, an international and intercontinental research context is indispensable.

### Limitations

This evaluation of quality of information on the Internet about rare diseases is based on information websites available in the German language and/or hosted in Germany, Austria, and Switzerland. Information available on social media accounts were not included in the analysis [34]. The quality criteria cannot verify the actual medical content of health information. These criteria simply verify the factors influencing good thematic content, as well as the quality of the website itself. An evaluation of the quality of information about specific disease groups (eg, rare skin diseases) is not feasible due to the ambiguous classification of rare diseases provided by Orphanet.

### Conclusions

The quality of information on the Internet about rare diseases was assessed based on 13 quality criteria for websites providing medical information about rare diseases. Overall, the quality of information on the Internet about rare diseases is insufficient, quality certificates are rarely used, and important quality criteria are often not fulfilled. Subgroup analyses have shown that information provided by support groups and patient organizations are as reliable as information provided by medical institutions. Additionally, there are some information categories that are underrepresented (eg, information about psychosocial counseling, social-legal advice, and family planning). These information categories need to be strongly addressed in future research on information on websites. Nevertheless, this study has shown that support groups are extremely important for patients suffering from a rare disease and their relatives.

## Abbreviations

ACHSE eV: German Alliance of Chronic Rare Diseases
afgis: German Action Forum Health Information System
HONcode: Health On the Net Foundation Code of Conduct
